# Nomogram to predict FSH starting dose in poor ovarian response women in progestin primed ovarian stimulation protocol

**DOI:** 10.1186/s12905-023-02327-x

**Published:** 2023-04-28

**Authors:** Shuxie Wu, Yanping Li, Gao Wu, Hanbin Wu

**Affiliations:** 1 Hospital of Obstetrics and Gynecology, Fudan University, Shanghai, 200080 China; 2 Reproductive Medicine Center, Xiangya Hospital, Central South University, Changsha, 410000 China; 3 Department of Pharmacy, First Affiliated Hospital of Naval Military Medical University, Shanghai, 200081 China; 4 Clinical Pharmacy, Shanghai East Hospital, Tongji University School of Medicine, 150 Jimo Road, Shanghai, 200120 China

**Keywords:** Nomogram, Starting dose, Poor ovarian response, Progestin-primed ovarian stimulation, Follicle-stimulating hormone

## Abstract

Prediction of individual ovarian response to exogenous gonadotropin is a cornerstone for success and safety in all controlled ovarian stimulation (COS) protocols. Providing the best FSH starting dose according to each woman’s own characteristics is the key to the success of individualized treatment. The objective of this investigation was to evaluate the potential application of a novel nomogram based on antral follicle counting (AFC), anti-Müllerian hormone (AMH) and body mass index (BMI) as a tool to optimize the follicle-stimulating hormone (FSH) starting dose in women with poor ovarian response in in-vitro fertilization (IVF)/intra-cytoplasmic sperm injection (ICSI) cycles in progestin-primed ovarian stimulation (PPOS). We performed a retrospective analysis involving 130 poor ovarian responders undergoing IVF/ICSI cycles in a PPOS protocol from June 2017 to February 2019 in our reproductive center. The individual FSH starting dose was selected according to patients’ clinical history and characteristics. The influence of variables including age, BMI, AMH and AFC on the FSH starting dose was assessed through multiple regression analysis. We used the variables reaching the statistical significance for calculation for the final predictive model. In the univariate analysis, BMI, AMH and AFC were significant (P < 0.05) predictors of FSH starting dose, age was canceled. In the multivariate analysis, BMI, AMH and AFC remained significant (P < 0.05). According to the nomogram, 118 patients (90.77% of 130) would have received a higher FSH starting dose and 12 patients (9.23% of 130) a lower FSH starting dose than practice dose. The application of the nomogram based on three variables easily determined in clinical practice: BMI, AMH and AFC would lead to a more tailored FSH starting dose in women with poor ovarian response.

## Introduction

The development of the basic theory of folliculogenesis and the improvement of embryo vitrification and frozen-thawed embryo transfer (FET) technology in reproductive medicine have given assisted reproduction technology (ART) clinicians an opportunity to consider a new strategy of using progestin (P) as an effective alternative to gonadotrophin-releasing hormone (GnRH) analogues for improving practices and results of in vitro fertilization (IVF) attempts [1–5].

The new stimulation protocol named progestin-primed ovarian stimulation (PPOS) created by Dr. Kuang using exogenous progestin (progesterone) negative feedback on the hypothalamus-pituitary-ovarian axis (HPOA) to inhibit the synthesis and secretion of pituitary luteinizing hormone (LH) [6–8]. PPOS protocol can improve ovarian response, prevent premature LH surge, increase the number of transplantable embryos and reduce the cycle cancellation [9]. The PPOS is not only effective for patients with a normal ovarian response, but has also been effectively applied in aged patients and those with poor ovarian response (POR) [9–11].

Prediction of individual ovarian response to exogenous gonadotropin is a cornerstone for success and safety in all controlled ovarian stimulation (COS) protocols. Ovarian stimulation protocols should avoid the development of poor or excessive ovarian response, which may lead to cycle cancellation or an increased risk of ovarian hyperstimulation syndrome (OHSS) [12]. In recent years, the concept of “one size fits all” has evolved into a concept of “individualization” in IVF [13]. The main objective of the individual treatment in FSH starting dose and protocol is to offer every single women the best treatment strategy based on her own characteristics thus allowing a high chance of success and of course minimizing the risk of cycle cancellation and ovarian hyperstimulation syndrome.

Therefore, the selection of the FSH starting dose is one of the most important clinical decision and it is fundamental for IVF outcomes [14]. There is no established criteria on how to select the proper starting dose of FSH for patients with POR. Usually reproductive specialists choose the FSH starting dose according to knowledge of their patients and established clinical practice to evaluate of the clinical and hormonal profile of the patient before and during ovarian stimulation, and counterbalance risks and benefits [15].

POR usually indicates a reduction in follicular response, resulting in a reduced number of retrieved oocytes and poor embryo quality. The management of POR is a frustrating event for both patients and clinicians which is associated with high cycle cancellation rate and poor pregnancy outcomes [16, 17]. Although different types of COS regimens have been used to improve the reproductive outcomes in patients of POR, there is still no consensus on the ideal COS regimens in such patients [18]. Several previous studies have shown that the PPOS protocol may be a better regime for POR patients which could effectively improve clinical pregnancy rate and live birth rates [2, 10].

Recently, nomogram has been elaborated to calculate the most appropriate FSH starting dose in IVF cycles [19–21]. The nomogram may be the basis for the individualization of the FSH starting dose for patients with normal response and could help to make more objective and quantitative prediction according to ovarian response. However, these nomograms were developed for normal ovarian response only, there was no nomogram to calculate proper FSH starting dose for POR patients. For this reason, we wanted to investigate a nomogram for POR patients to calculate appropriate FSH starting dose in the PPOS protocol, based on a comprehensive consideration of BMI and several ovarian reserve tests [14], such as serum levels of AMH or FSH and AFC.

## Materials and methods

### Study Population

A retrospective analysis study was conducted at the Reproductive Medicine Center, Xiangya Hospital, Central South University, Changsha, China. We reviewed the electronic medical records of patients undergoing IVF-FET between June 2017 and February 2019 in our fertility central. The database contained clinical and laboratory information on IVF/ ICSI treatment cycles.

Poor ovarian response was defined according to the Bologna criteria [9] and existence of at least two of the following criteria: (1) a previous history of POR (retrieved oocytes ≤ 3) in a conventional stimulation protocol, (2) advanced maternal age (≥ 40 years) or any other risk factors for POR (e.g. a history of ovarian surgery) and (3) abnormal ovarian reserve test (i.e. antral follicle count (AFC) < 5 follicles or anti-Mullerian hormone (AMH) < 1.1 ng/ml).

**Fig. 1:**
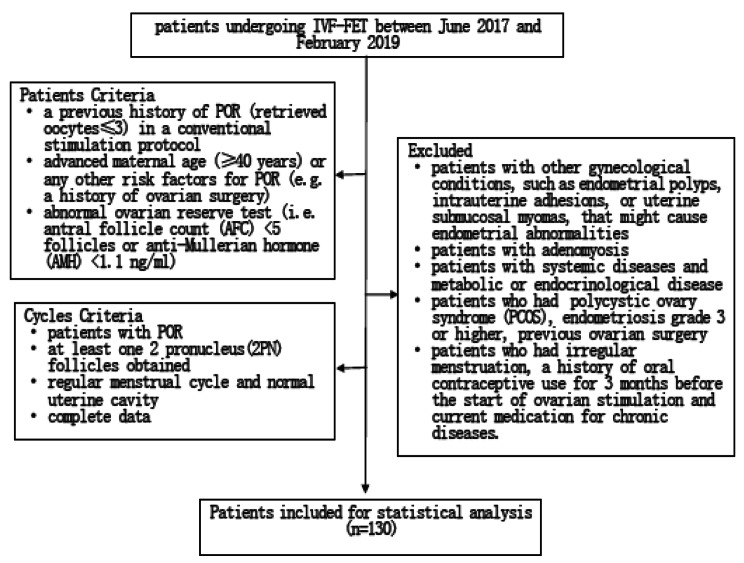
Patients Selection Flowchart

Cycles were selected for analysis if all the following inclusion criteria were satisfied: (1) patients with POR; (2) at least one 2 pronucleus(2PN) follicles obtained; (3) regular menstrual cycle and normal uterine cavity; (4) complete data. The exclusion criteria were as follows: (1) patients with other gynecological conditions, such as endometrial polyps, intrauterine adhesions, or uterine submucosal myomas, that might cause endometrial abnormalities; (2) patients with adenomyosis; (3) patients with systemic diseases and metabolic or endocrinological disease; (4) patients who had polycystic ovary syndrome (PCOS), endometriosis grade 3 or higher, previous ovarian surgery; (5) patients who had irregular menstruation, a history of oral contraceptive use for 3 months before the start of ovarian stimulation and current medication for chronic diseases (Flowchart see Fig. 1).

### Study procedures

All patients were administered with FSH (150–300 IU/d; Livzon Pharmaceutical Group Inc., China), and human menopausal gonadotropin (HMG) (75–150 IU/d, Livzon Pharmaceutical Group Inc., China) and medroxyprogesterone acetate (MPA) (10 mg/d, Shanghai Xinyi Pharmaceutical Co., China) from three-day cycle onward, and the empiric choice of the dose was according to the patient’s AFC and BMI. These examinations were performed every 2–4 days to record the numbers and diameters of the developing follicles. The serum FSH, LH, oestradiol (E2), and progesterone (P) concentrations were measured on the same days as the ultrasound examinations. When a dominant follicles reached 18 mm or two dominant follicles reached 16 mm in diameter, the final stage of oocyte maturation was triggered by using triptorelin (0.1 mg; GenScience Pharmaceutical Co.,Ltd., China ) and Human chorionic gonadotropin (HCG) (2,000 IU; Livzon Pharmaceutical Group Inc., China). Transvaginal ultrasound–guided oocyte retrieval was performed 34–36 h after the trigger. All follicles with diameters greater than 10 mm were aspirated.

The aspirated oocytes were fertilized in vitro by either conventional insemination or ICSI. According to the criteria described by Zhao et al. [22] high-quality embryos were frozen by vitrification on the 3rd day following oocyte retrieval, and low-quality embryos were placed in extended culture. Subsequently, blastocysts with good morphological grades were frozen on day 3 of culture. The procedure of freezing and thawing cleavage-stage embryos and blastocysts was routinely performed according to Kuang’s method [10].

### Statistical analysis

The average dose throughout the cycle was considered as the optimal starting dose which was related to the number of BMI, AMH, AFC and age and a multivariable linear regression model was applied to predict the quantity of optimal starting dose to obtain the desired response. To screen variables, unary linear regression was used with T-test and statistical significance was set for p < 0.05. All statistical analyses were done and nomograms was produced by R-3.5.2. For the present analysis, all parameters were analyzed as continuous variables and were reported as mean ± SD.

## Results

In total, 130 women were available for statistical analysis. Characteristics of patients and IVF cycles are reported in Table 1. Mean (± SD) age of patients was 37.79(± 5.65) years(range: 21–46). Mean (± SD) BMI of patients was 22.38 (± 2.14) kg/m^2^ (range: 17.2–24.5). Mean (± SD) retrieved oocytes of patients was 3.04 (± 1.79) (range: 1–12). Mean (± SD) serum AMH of patients was 0.43 (± 0.32) ng/ml (range: 0.06–2.08). Mean (± SD) AFC of patients was 3.06 (± 1.73) (range: 0–6). Mean (± SD) number of two pronucleus(2PN) zygotes of patients was 2.21(± 1.46) (range: 1–9).

**Table 1:** Demographic characteristics of patients in the study

Variables	Mean ± SD
Age, years	37.79(± 5.65)
BMI (kg/m2)	22.38 (± 2.14)
**Retrieved oocytes**	**3.04 (± 1.79)**
**Serum AMH (ng/mL)**	**0.43 (± 0.32)**
**Antral follicle count**	**3.06 (± 1.73)**
**No. of 2PN**	**2.21(± 1.46)**

**Fig. 2:**
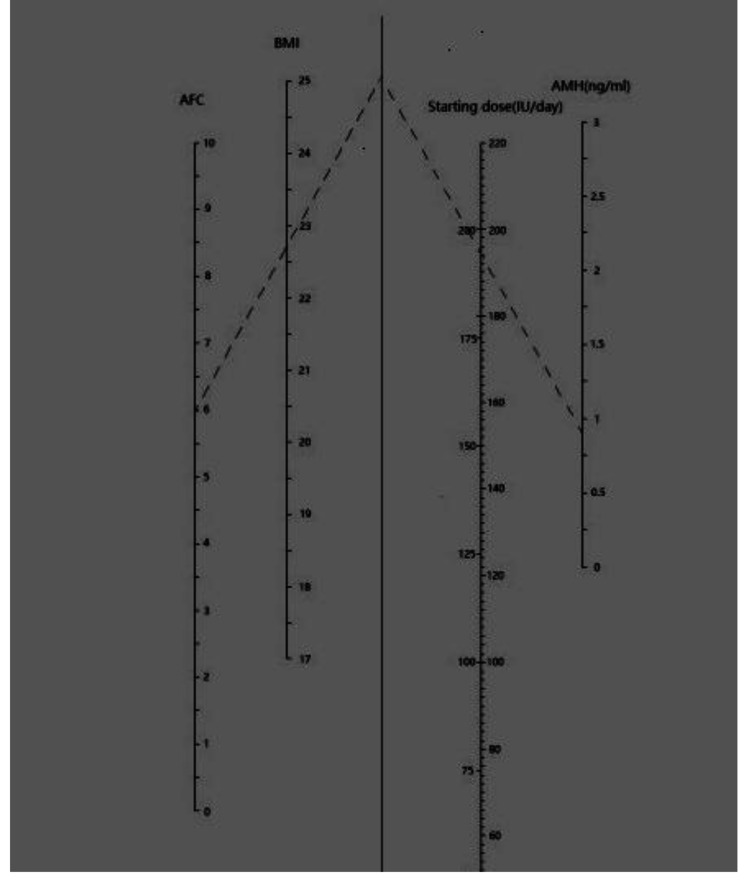
The nomogram for the calculation of the FSH starting dose based on BMI, AFC and AMH. In the example, for BMI = 22.6 patient with AFC = 6 and AMH = 0.8(ng/mL), the FSH starting dose is 194 IU. Since the dose was increased with 1/4amp steps (18.75 IU), on the right side of the FSH starting dose column, the FSH dose as selected is reported (206.25 IU for the example)

Results of univariate and multivariate regression analyses with the starting dose of FSH (average dose of FSH of whole cycle) as a dependent variable are shown in Table 2. The independent variables were: age, BMI, AMH and AFC. The univariate regression analysis showed that the starting dose of FSH was significantly predicted by BMI, AMH and AFC, age was canceled because of p > 0.05. In the multivariate regression analysis the statistical significance was reached only for BMI, AMH and AFC. The prediction model was constructed using the combinations of three dependent variables and had the best goodness of fit. According to the model shown in Table 2, the nomogram to elaborate a starting dose was generated (Fig. 2).

**Table 2:** Predictors of the starting dose of FSH in univariate and multivariate backward regression analysis

Variables	Univariate			Multivariate		
	Regression coefficient	Standard Error	P	Regression coefficient	Standard Error	P
age	0.7103	0.4948	0.144			
BMI	2.757	1.161	0.03933	2.757	1.161	0.01887
AMH	10.965	4.074	0.02321	7.857	3.947	0.04843
AFC	2.642	1.034	0.01132	2.746	1.052	0.00996

Figure 2 The nomogram for the calculation of the FSH starting dose based on BMI, AFC and AMH. In the example, for BMI = 22.6 patient with AFC = 6 and AMH = 0.8(ng/mL), the FSH starting dose is 194 IU. Since the dose was increased with 1/4amp steps (18.75 IU), on the right side of the FSH starting dose column, the FSH dose as selected is reported (206.25 IU for the example).

**Fig. 3:**
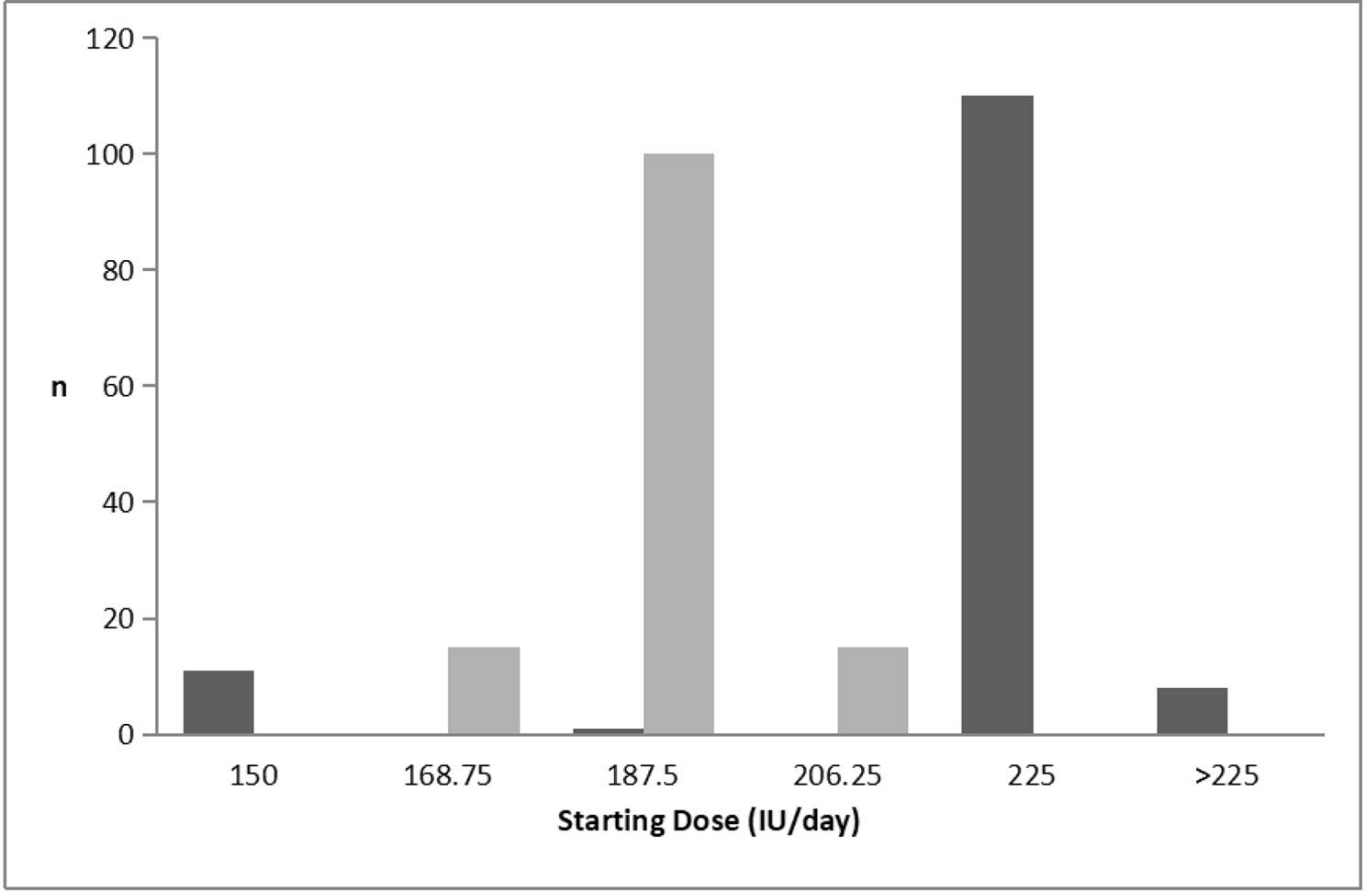
Distribution of FSH starting dose (IU). Black as actually prescribed by clinicians, gary as it has been calculated by the nomogram

The three-variables-nomogram reported in Fig. 1 was then applied on the same population on which it was calculated (n = 130). 118 of patients (90.77% of 130) would have received a higher FSH starting dose and 12 of patients (9.23% of 130) would have received a lower one than practice dose managed according to the nomogram. The different distribution of the FSH starting dose between actually prescribed and calculated by the nomogram is reported in Fig. 3. As shown, in actual prescription, n = 11/130 (8.46%) of patients received a starting dose of 150 IU,n = 1/130 (0.77%) of patients received a starting dose of 187.15 IU, n = 110/130 (85.62%) of patients received a starting dose of 225IU, while n = 8 /130 (6.15%) of patients were treated with starting dose more than 225 IU. According to the nomogram, the predicted an FSH starting dose mainly concentrated on two doses,15/130 (11.54%), 100/130 (76.92%) and 15/130 (11.54%) of patients received a starting dose of 168.75IU, 187.5 IU and 206.25 IU respectively.

Figure 3 Distribution of FSH starting dose (IU). Black as actually prescribed by clinicians, gary as it has been calculated by the nomogram.

## Discussion

The present study represents the first study to establish nomograms that predict the number of 2PN zygotes for the personalization of the FSH starting dose in women with poor ovarian response undergoing IVF treatment with PPOS by using several combinations including BMI, AMH level, and AFC. The nomogram we developed may be clinically useful and seems a promising tool to individualize the treatment upon ovarian reserve in poor ovarian responders reducing the inter-operator variability derived from clinicians’ clinical experience during their daily clinical practice.

The main characteristic of the PPOS protocol is the use of progesterone for example medroxyprogesterone acetate (MPA) to block the rise of LH to produce more follicles during the follicular phase [4, 7]. The PPOS protocol breaks away from the convention of inhibiting LH surge in the traditional down-regulation protocol of relying on Gonadotropin releasing hormone (GnRH) analogues. Because of the asynchrony between the endometrium and embryo with PPOS stimulation, all the oocytes/embryos should be cryopreserved for later transfer [2]. There is also some evidence that PPOS protocol produced more mature oocytes and embryos for cryopreservation and achieved high-quality oocytes and satisfactory pregnancy outcomes [7, 32]. In this trial, we included patients with abnormal ovarian reserve test ( with AFC < 5 follicles or AMH < 1.1 ng/ml). The criteria for this trial were under the widely accepted Bologna criteria. These POR patients provide a good model for investigating better clinical outcome of PPOS. Different types of therapeutic approaches have been reported to improve the cycle outcomes in patients with poor ovarian response, but still there is no consensus on one stimulation protocol with compelling advantage over another. Several reports confirm that the cycle outcomes of patients with POR cannot improve by increasing gonadotropin doses, as the assumption that number of oocytes retrieved may be a function of daily gonadotropin dose is controversial. The possible etiologies of POR could be explained by limited ability to recruit a wave of follicles and different sensitivity of early antral follicles to FSH [23–26]. Studies have shown that poor responder patients were able to recruit an extra oocyte or two by increasing the exogenous FSH dose, with the possible improvement in IVF outcome [27]. However, mature oocyte may determine the outcomes of IVF, not the number of retrieved oocyte. A study demonstrated that a significantly higher rate of immature oocytes after COS in women over forty years-of-age as compared to younger women [2, 28]. Recently, a prospective controlled study had shown that PPOS protocols can overcome premature ovulation and not adversely affect the quality of oocytes for poor responders. The profound suppression of endogenous FSH and LH at the stage of follicular recruitment could be avoided during PPOS, then a better egg retrieval could be obtained [7, 9, 11]. Meanwhile, PPOS can induce an increase in basal plasma FSH by using hMG and FSH surge occurred on the trigger day. The FSH surge before ovulation can promote the development of the follicles and effectively improve the developmental potential of follicles [1]. The clinical study by Huang showed that the MII oocyte, fertilization, and high-quality embryo rates in the PPOS group were significantly higher than those in the antagonist group (p < 0.05) [11]. Therefore, the PPOS protocol may be a better ovarian stimulation regime for poor responder patients. The main problem of POR was not only the depletion of the oocytes but the decrease in oocyte quality. The controlled ovarian hyperstimulation generally results in unsatisfactory oocyte yield for POR during IVF-ET cycles. Moreover, the different sensitivity of early antral follicles to FSH led follicles to develop non-synchronously in different timing of COS with PPOS. It is critical to obtain more high-quality oocytes for later embryo transfer in POR patients [29]. Even if POR patients go forward with very few follicles, the quality of oocyte retrieved is high enough, so that fertilization and embryo transfer will be successful. On the contrary, amounts of retrieved oocytes with lots of which are discarded because of their poor-quality will lead to a bad result [30]. A major problem of the poor responder patients not only is the fewer oocytes recovered number but the oocyte quality is diminished, for these patients produce unsatisfactory oocyte during IVF cycles [29]. Previous studies confirmed that the fertilization rate (2PN) was not affected by the increasing age of the women in IVF or ICSI. This means that 2PN has less variation in IVF procedures [31, 32]. Therefore, we selected 2PN zygotes as the criterion for successful oocytes retrieval in the present study.

The majority of reproductive specialists agree that nowadays AMH and AFC are considered as two reliable markers for predicting ovarian response [13]. Indeed AMH and AFC have a strong correlation and are actually measure the same thing: ovarian reserve [14]. AMH is produced exclusively by granulosa cells of the developing preantral and antral follicles and reflects the overall amount of the granulosa cells and is thought to be a direct surrogate of the number of growing follicles in follicular pool. Serum AMH level does not exhibit significant intra- and inter-cycle variability and has the advantage of its operator-independence. Serum AMH level declines by 5.6% per year and is affected by factors such as follicle size, granulosa cell volume and genetic characteristics. AMH has low sensitivity and specificity for the prediction of successful achievement of pregnancy in IVF [33, 34]. AFC much more immediately represents the number of growing primordial follicles remaining in the ovary and reflects the ovarian follicular patrimony. AFC has a great advantage that it could be detected at the same moment in which clinicians examine the patient and an inherent shortcoming of high inter-observed variability. Although AFC has a high specificity for predicting a poor response, its sensitivity is low [12, 35]. BMI was another significant parameter predicting the FSH starting dose. It is known today that weight and body mass are a highrisk factor for menstrual dysfunction and anovulation. Obese women have a lower chance of conception following assisted reproductive techniques (ART). Obesity has a negative impact on the woman´s reproductive system through various pathways, including impaired ovarian, follicular development, quantitative and qualitative development of the oocyte, fertilization, and embryo development and implantation. Increased BMI significantly reduces the chance of clinical pregnancy in IVF [36]. Obese women require higher amounts of gonadotropins respond poorly to ovarian stimulation and more days to achieve follicular maturation. It is well documented that increased BMI is associated with low oocyte retrieval and poor embryo quality [37, 38]. The influence of age, AMH level, AFC level and BMI on the FSH starting dose was examined through multiple regression analysis in present study. our study demonstrated no relationship between age and FSH starting dose of POR patients. AMH level, AFC and BMI were important parameters to predict ovarian response to exogenous FSH.

Clinicians could assess markers of ovarian reserve and choose the best therapy in order to improve IVF outcomes. The ovarian reserve markers recently used include age, BMI, FSH, AMH and AFC et al. [22]. One single ovarian reserve marker may be insufficient to predict ovarian response to exogenous FSH, a combination of multiple markers may improve the accuracy in predicting ovarian response to gonadotropins to optimize the FSH starting dose in IVF/ICSI cycles at individual level [15, 22]. Even though it is well-recognized that the female reproductive capacity is decreasing with the age increasing and the prevalence of POR increases with age, the prevalence of dominant follicles does not differ with age and women’s age has no impact on the summed score for cleavage stage embryo quality [9, 13, 31]. The previous findings shown that the fertilization rate (2PN) was not affected by women’s age in IVF or ICSI [31]. The most frequent cause of poor ovarian response may be diminished ovarian reserve in both older women and younger women [10]. Prediction of starting dose had no concern with age in patients with poor ovarian response in the present study. The reason may be that the number of young patients in our study is too small to be statistically significant. Our data analysis showed that the main FSH starting dose prescribed on clinical experience was 225 IU (41.22%), while the nomogram-based prescription of FSH starting dose was mainly either 187.5 IU or 206.25 IU (total percentage of the two was 88.52%). This suggests that when faced with patients with poor ovarian response, our doctors often worried that the initial dose will not be sufficient, and gradually reduce it at a later stage. But according to the nomogram, the starting dose can be reduced to decrease the financial burden and risk of side effects in patients. As the application of the nomogram could lead to more accurate starting dose.

Our study does have limitations. Firstly, this is a retrospective study and its small sample size for a study population treated in a single centre. The second limitation is that our findings are no follow up outcme implantation and pregnancy rate and not be generalizable to all FET cycles. The main strengths of this study include the good pregnancy rate and lack of cases of OHSS are important. Nonetheless, additional larger studies to establish the safety and efficacy of this standardized treatment protocol would support the predicting protocol presented here.

In conclusion, the individual FSH starting threshold dose for ovulation induction in patients with poor ovarian response can be predicted based on easily available three variables detected in clinical practice: AMH, AFC and BMI. An FSH dosage nomogram to predict the FSH starting dose was constructed based on these predictive factors.

## Data Availability

The datasets used and/or analysed during the current study available from the corresponding author on reasonable request.

## Abbreviations

COS: controlled ovarian stimulation
AFC: antral follicle counting
AMH: anti-Müllerian hormone
BMI: body mass index
FSH: follicle-stimulating hormone
IVF: in-vitro fertilization
ICSI: intra-cytoplasmic sperm injection
PPOS: progestin-primed ovarian stimulation
FET: frozen-thawed embryo transfer
ART: assisted reproduction technology
P: progestin
GnRH: gonadotrophin-releasing hormone
POR: poor ovarian response
HPOA: hypothalamus-pituitary-ovarian axis
OHSS: ovarian hyperstimulation syndrome
2PN: 2 pronucleus
